# Addressing vaccine hesitancy and access barriers to achieve persistent progress in Israel’s COVID-19 vaccination program

**DOI:** 10.1186/s13584-021-00481-x

**Published:** 2021-08-02

**Authors:** Bruce Rosen, Ruth Waitzberg, Avi Israeli, Michael Hartal, Nadav Davidovitch

**Affiliations:** 1 Myers-JDC-Brookdale Institute, JDC Hill, Jerusalem, Israel; 2 Hebrew University Paul Baerwald School of Social Work and Social Welfare, Jerusalem, Israel; 3 Department of Health Care Management, Faculty of Economics & Management, Technical University Berlin, Berlin, Germany; 4 Hebrew University Hadassah Medical School, Jerusalem, Israel; 5 Ministry of Health, Jerusalem, Israel; 6 Faculty of Health Sciences, Ben-Gurion University of the Negev, School of Public Health, Beersheba, Israel; 7 Taub Center for Social Policy Studies in Israel, Jerusalem, Israel

## Abstract

As of March 31, 2021, Israel had administered 116 doses of vaccine for COVID-19 per 100 population (of any age) – far more than any other OECD country. It was also ahead of other OECD countries in terms of the share of the population that had received at least one vaccination (61%) and the share that had been fully vaccinated (55%). Among Israelis aged 16 and over, the comparable figures were 81 and 74%, respectively. In light of this, the objectives of this article are:

1. To describe and analyze the vaccination uptake through the end of March 2021
2. To identify behavioral and other barriers that likely affected desire or ability to be vaccinated
3. To describe the efforts undertaken to overcome those barriers

Israel’s vaccination campaign was launched on December 20, and within 2.5 weeks, 20% of Israelis had received their first dose. Afterwards, the pace slowed. It took an additional 4 weeks to increase from 20 to 40% and yet another 6 weeks to increase from 40 to 60%. Initially, uptake was low among young adults, and two religious/cultural minority groups - ultra-Orthodox Jews and Israeli Arabs, but their uptake increased markedly over time.

In the first quarter of 2021, Israel had to enhance access to the vaccine, address a moderate amount of vaccine hesitancy in its general population, and also address more intense pockets of vaccine hesitancy among young adults and religious/cultural minority groups. A continued high rate of infection during the months of February and March, despite broad vaccination coverage at the time, created confusion about vaccine effectiveness, which in turn contributed to vaccine hesitancy. Among Israeli Arabs, some residents of smaller villages encountered difficulties in reaching vaccination sites, and that also slowed the rate of vaccination.

The challenges were addressed via a mix of messaging, incentives, extensions to the initial vaccine delivery system, and other measures. Many of the measures addressed the general population, while others were targeted at subgroups with below-average vaccination rates. Once the early adopters had been vaccinated, it took hard, creative work to increase population coverage from 40 to 60% and beyond.

Significantly, some of the capacities and strategies that helped Israel address vaccine hesitancy and geographic access barriers are different from those that enabled it to procure, distribute and administer the vaccines. Some of these strategies are likely to be relevant to other countries as they progress from the challenges of securing an adequate vaccine supply and streamlining distribution to the challenge of encouraging vaccine uptake.

**Supplementary Information:**

The online version contains supplementary material available at 10.1186/s13584-021-00481-x.

## Background

As of the end of 2020, the State of Israel had administered almost 11.0 doses of vaccine per 100 population (all ages), while the next highest rates were 3.5 in Bahrain and 1.4 in the United Kingdom. At that time, all other countries had administered less than 1 dose per 100 population. As we described in a recent article [1], numerous factors contributed to this early success and they are listed in Table 1. They can be divided into three major groups: long-standing characteristics of Israel that are extrinsic to health care, long-standing characteristics that are health-system specific, and more recent factors that are specific to the COVID-19 vaccination effort. Subsequently, in a series of commentaries on the early Israeli experience, several authors have identified reproducible “take-away” ideas and lessons for the United States [2–4], Canada [5], the United Kingdom [6], and other countries more generally [7, 8].

**Table 1:** Factors that contributed to the early success of Israel’s vaccination effort

**A. Long-standing characteristics of Israel that are extrinsic to health care**	
1. Israel’s small size, in terms of both area and population	
2. Israel’s centralized national system of government	
3. Israel’s experience in, and infrastructure for, planning and implementing prompt responses to large-scale national emergencies	
**B. Long-standing characteristics that are health-system specific**	
4. The organizational, IT and logistic capacities of Israel’s community-based healthcare providers	
5. The availability of a cadre of well-trained, salaried, community-based nurses who are employed directly by the health plans	
6. The tradition of effective cooperation between government, health plans, hospitals, and emergency care providers – particularly during national emergencies – and the frameworks for facilitating that cooperation	
7. The existence of well-functioning frameworks for making decisions about vaccinations and support tools for assisting in the implementation of vaccination campaigns	
**C. More recent factors that are specific to the COVID-19 vaccination effort**	
8. The rapid mobilization of special government funding for vaccine purchase and distribution	
9. Timely contracting for a large amount of vaccines relative to Israel’s population	
10. The use of simple, clear and easily implementable criteria for determining who had priority for receiving vaccines in the early phases of the distribution process	
11. A creative technical response that addressed the demanding cold storage requirements of the Pfizer-BioNTech COVID-19 vaccine	
12. Initial outreach efforts	

Source: [1]

In this article we consider developments in the national vaccination program though the end of the first quarter of 2021.

As of March 31, 2021 Israel continued to be ahead of other OECD countries with 116 doses per 100 population of all ages [9]. Israel was also ahead of other OECD countries in terms of the share of the population that had received at least one vaccine dose (61%) and the share that had been fully vaccinated^1^ (55%). These results are even more striking when one focuses on the vaccine-eligible population, as the vaccine had been authorized only for people aged 16 and over,^2^ and Israel has a relatively high proportion of population under age 16.^3^ Among Israelis aged 16 and over, 81% had received a first dose by March 31 [11].

Although the first several weeks of Israel’s vaccination effort went quite smoothly and speedily, in the subsequent weeks and months the vaccination effort encountered several challenges, which reduced the pace of vaccine uptake. In this paper we provide an overview of those challenges and analyze what Israel has done, and continues to do, to address them. We hope that the analysis will be informative and useful for other countries, as they proceed with their own vaccination campaigns.

Implementing any successful vaccination campaign entails at least three major factors: having enough doses of an effective vaccine, having the logistical and workforce capacity to deliver those doses to the population, and having a population willing to get vaccinated [12].

In the months before Israel’s vaccination campaign, and during the early stages of that campaign, there were many unvaccinated individuals eager to get vaccinated. This enabled Israel to focus effectively on acquiring a sufficient stock of vaccines and putting into place, as well as continuing to refine, an effective delivery system. This created a certain momentum, with many Israelis eagerly lining up for vaccinations and creating a bandwagon effect. Subsequently, Israel had to give greater attention to the extent to which the remainder of the population was interested in being vaccinated.

Throughout the first quarter of 2021, Israel continued to benefit from many of the factors that had contributed to its successful initial rollout in late December 2020. It continued to expand its supply of Pfizer vaccines, in part through a collaboration agreement between the Government of Israel and Pfizer, Inc. Israel committed to provide anonymized, aggregated epidemiological data about the vaccination and health status of its residents, while Pfizer agreed to continue to provide Israel with enough doses to vaccinate its entire adult population.

In parallel with the stable vaccine supply, Israel, through a series of steps, expanded the age groups eligible for the vaccine. Israel continued to rely on its four health plans as the main deliverers of the vaccine. These plans provide health care coverage for the entire population and have outstanding logistical and IT capacities. Independent Israeli news media continued to highlight the high level of demand for vaccines and the satisfaction of those vaccinated. In addition, Israel’s government, media, and health care leaders promoted national pride in Israel being ahead of other countries in vaccination coverage.

Eleven of the twelve factors listed in Table 1 as facilitating the success of the early phase of Israel’s vaccination campaign, were primarily relevant to securing an adequate supply of vaccines and delivering them, rather than encouraging uptake. Ultimately, however, those measures proved insufficient to ensure sustained rapid uptake of the vaccine over time, and Israel had to adopt additional measures.

This article has three objectives:

1. To describe and analyze the vaccination uptake through the end of March 2021.
2. To identify behavioral and other barriers that likely affected desire or ability to be vaccinated.
3. To describe the efforts undertaken to overcome those barriers.

The findings section is structured along those three objectives. It begins with an analysis of the changes over time in the pace of vaccinations, based on age-specific data from the Israel Ministry of Health (as described in Appendix. The second part of the findings section explores the barriers to vaccine uptake, while the third part documents the efforts to overcome the barriers. The latter two sections are based on journal articles, unpublished government reports, newspaper accounts, discussions with experts and stakeholders. In all three parts of the findings sections, attention is given first to the Israeli population in its entirety, and this is followed by drill-downs related to specific age and ethnic/religious groups. The analysis of impediments to vaccination and how they were addressed is summarized in Table 2, while Table 3 provides a chronology of key related events.

**Table 2:** Overview of impediments to vaccination, population groups particularly affected, and key responses

Impediments	Population groups particularly affected by the impediment	Steps taken to address impediments
**Logistical impediments**		
Difficulty in reaching vaccination sites	Arabs, residents of remote areas (periphery), Bedouin	Mobile units
Need for child care during vaccination	Arabs; ultra-Orthodox	Coordinators appointed
**Perceptual/behavioral impediments**		
Reticence to adopt a new product; uncertainty among the part of the public about benefits of this vaccine	All except early adopters and high risk groups	Initiating program only after FDA approval; dissemination of evidence of effectiveness; passage of time; examples set by public figures and celebrities
General concern about known/possible side effects	All groups	Dissemination of evidence of limited side effects, tailored messages, passage of time and seeing that those who received the vaccine are ok
Concern about adverse effects on fertility / pregnancy	Arabs; ultra-Orthodox; young adults	Dissemination of information on COVID-19 risks to pregnancy; scientist reassurance re: fertility, media coverage of cases where pregnant women got severely ill and were not vaccinated.
Confusing epidemiologic developments in Israel	All groups	Provision of explanations for confusing developments; dissemination of information from new micro- and macro-level studies
Perception of limited personal benefits from vaccine	Young adults	Mobile units in places where they frequent (universities, beaches, commercial streets), campaigns.
Limited trust in authorities and vaccine safety	Arabs and some ultra-Orthodox Jewish groups	Partnering with community leaders
Language and communication barriers	Arabs	Development of Arabic-language materials and use of Arabic-language media
**Diverse and cross-cutting impediments**		
Inertia; perception that vaccination costs/risks outweighed potential benefits	Entire population	Establishment of the Green Pass program and other incentives
Unique needs and concerns of culturally-defined population groups	Arabs and ultra-Orthodox	Establishment of dedicated task forces for Arab and ultra-Orthodox populations; tailored messaging

**Table 3:** Chronology

Date	Event
December 20	Vaccination campaign launched, covering ages 60+ and additional priority groups
December 27	Third lockdown imposed
January 7	Age eligibility extended from 60+ to 55+
January 7	Lockdown tightened
Mid-January	Shift from large vaccination centers to health plan clinics
January 19	Age eligibility extended to 40+
January 23	Ages 16–18 made eligible
January 26	Complete closure of borders
February 2	Age eligibility extended to 20+
February 7	End of lockdown
February 21	Green Pass instituted
February 21	Phase 1 relaxation of community restrictions
March 19	Phase 2 relaxation of community restrictions

**Sources:** [11, 13]

In light of the achievements of Israel’s vaccination program and the diversity of its population, Israel’s experiences should be of interest and benefit to many other nations. At the same time, it is important to keep in mind that cross-national learning is complicated by differences in health care systems and beyond. Table 4 provides a brief overview of Israeli health care, while a discussion of how Israel differs from other countries can be found in our earlier article on the first phase of Israel’s vaccination rollout (1).

**Table 4:** Overview of Israeli health care

Israel’s Ministry of Health is responsible for the governance of the health system overseeing the performance of hospitals, health plans, and health care professionals. The MoH is responsible for providing a broad range of public health services. Israeli health care is regulated by a national health insurance (NHI) law, which ensures universal access to health services for all residents of Israel. Each resident is free to choose from among four competing non-profit health plans. The health plans are financed by government within the framework of NHI, and they are obligated to provide their members with a broad government-determined benefits package, which includes hospital care, community-based care, and various preventive services. Some of these services are provided directly by the plans, while others are purchased by the plans, for their members, from other providers. The health plans have sophisticated electronic health record systems that integrate information across providers, and well-developed systems for communicating and sharing information with their members.	

## Findings

### Changes over time in the pace of vaccination

When the vaccination program was launched in late December 2020, eligibility was restricted to persons aged 60 or over, nursing home residents, people at high risk due to serious medical conditions, and front-line health care workers [1]. The range of eligible ages was then expanded periodically over the course of January and February. The government did not wait until all individuals from a certain age group got vaccinated to open the eligibility to the next group. When the number of people presenting for vaccinations declined, the authorities made an explicit decision that the health plans could begin scheduling vaccinations for the next priority group. As there was a large supply of vaccine, there were enough doses available for both the newer and the earlier priority groups [14] . Eligibility was extended to 55–59 year-olds on January 7; to 40–54 year-olds on January 19; and to 16–18 year-olds^4^ on January 23. On February 2, 19–39 year-olds became eligible for the COVID-19 vaccine so that, as of February 2, all Israelis aged 16+ were eligible [13]. On March 3, Israeli residents who had recovered from clinical COVID-19 became eligible for a single vaccine dose 3 months after their estimated infection date.

Because different age groups became eligible for vaccination at different dates, we present data on vaccination rates over time for both the entire population and selected age groups. We also present data separately for key population groups: ultra-Orthodox Jews, Israeli Arabs, and the general population.

As indicated in Table 5, at the end of 2020, the total population of Israel was 9.3 million. This included 1.2 million ultra-Orthodox Jews (13% of the total) and 1.9 million Arabs (21% of the total), so that the 6.2 million Jews who are not ultra-Orthodox (also referred to as the “general population”) accounted for 66% of the total population. The ultra-Orthodox and Arab populations have relatively high poverty rates and housing density levels, and relatively low rates of matriculation certification and employment.

**Table 5:** Selected indicators, by population group

	Total	General	ultra-Orthodox	Arab
Population, total, 2020	9,297,838	6,167,971	1,175,088	1,954,779
Percent of total population, 2020	100%	66%	13%	21%
Percent children, under age 18, 2020	33%	28%	54%	38%
Employment rate, males, ages 25–64, 2020	80%	86%	52%	69%
Employment rate, females, ages 25–64, 2020	74%	83%	78%	36%
Poverty rate for individuals*, 2016	22%	9%	53%	52%
Possession of matriculation certificates, 2016	69%	76%	54%	49%
Housing density for families with children, 2016	1.3	1.1	1.6	1.7

* In Israel, the poverty line is defined by the Social Security administration as 50% of the disposable median income (including transfer payments and after deduction of taxes), adjusted to the size of the family. The poverty rate for individuals reflects the number of individuals living in poor families

Sources:

The Haredi Institute for Public Affairs, The Quality of Life of Populations in Israel: Book of Tables, Data, and Charts. 2018. https://machon.org.il/publication/652/

The Haredi Institute for Public Affairs, Data Dashboard. https://machon.org.il/dashboards/

Central Bureau of Statistics, Labor Force Survey

#### Vaccination uptake for the entire population

**Figure 1:**
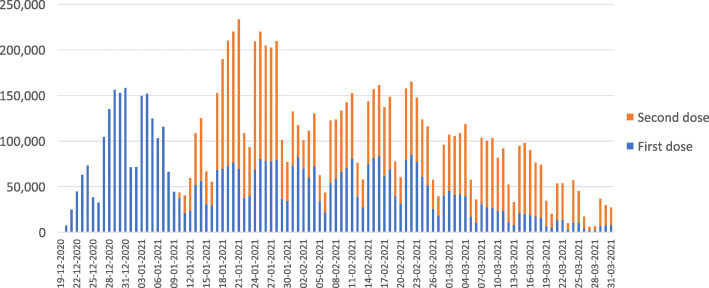
**Fig. 1** Number of Israelis vaccinated per day (December 19, 2020 – March 31, 2021)

As indicated in Fig. 1, between December 27 and January 7, on each weekday (i.e., excluding Fridays and Saturdays, which constitute the weekend in Israel^5^) over 100,000 Israelis (approximately 1.1% of Israel’s population of 9.3 million) received a first dose of the Pfizer vaccine. The number of daily first doses then went through a series of ups and downs but never again reached 100,000 [11].

Another way to look at the vaccination effort over time is to consider how long it took to increase the proportion of the population that received a first dose by 20 percentage points. Within 2.5 weeks of the December 20 launch (i.e., by January 7), 20% of Israelis had already received their first dose. Afterwards, progress continued, but the pace slowed dramatically. It took an additional 4 weeks to increase this proportion from 20 to 40%, and yet another 6 weeks for it to increase from 40 to 60%. This slowing rate of first dose uptake occurred despite the stepwise expansion of eligible age groups.

The slowdown in the vaccination pace may have had some indirect and unintentional positive effects, such as allowing health care professionals to allocate more of their time to other important health care tasks. However, in terms of the pandemic response, the slowdown in vaccinations delayed the acquisition of immunity for many individuals, impeded efforts to contain the pandemic, resulted in additional morbidity and mortality, and delayed the resumption of regular social and economic activity.

Supply constraints were not a major factor in the slowdown in the pace of first dose vaccinations. Israel had enough vaccine and enough delivery capacity (including wide geographic distribution of delivery sites) to continue administering first doses at the pace that prevailed during the initial weeks of the rollout.

Moreover, the administration of second doses, initiated three weeks after the launch of the vaccination campaign, played only a minor role in the slowdown in the pace of first doses. As can be seen in Fig. 1, in the latter half of January, when Israel began to administer second doses, the total number of daily doses (first + second) increased to over 200,000. During those two weeks, it may be that some of the slots that might otherwise have gone for first doses went instead to second doses. However, by February, the number of daily doses had dropped to below 150,000 on most days. The capacity to administer over 200,000 doses per day continued, both in terms of vaccine availability and personnel; the limiting factor was demand and uptake rather than supply.

Vaccine uptake in Israel was also not affected by the safety concerns regarding the AstraZeneca and Johnson & Johnson vaccines that appear to have affected uptake in many other countries. This is because Israel’s vaccination program relies on the Pfizer vaccine.

#### Vaccine uptake by age groups

**Figure 2:**
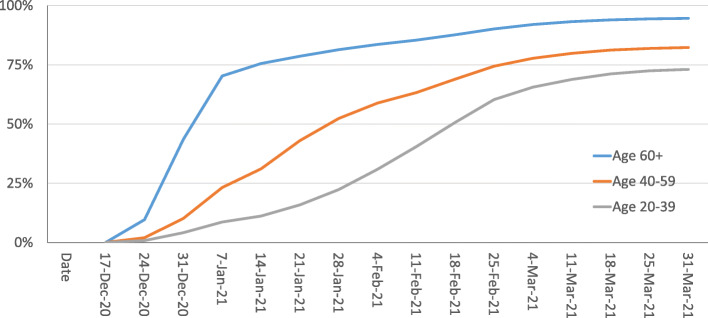
**Fig. 2** Vaccination uptake by age group, over time

Figure 2 presents weekly data on cumulative vaccination uptake, by age group, for Israelis aged 20+. Among persons aged 60 and over, who became eligible on December 20, the percentage who had received at least one dose increased from 0 to 70% during the first 3 weeks of eligibility, while it took another 3 weeks to reach 80% and a further 4 weeks to reach 90%.

The expansion of eligibility to additional age groups appears to have played only a minor role in the slowdown in the pace of vaccinations for the 60+ age group. Throughout most of the first quarter of 2021, Israel was not making full use of its vaccination capacity. Moreover, at both government and health plan levels, there was a clear commitment to giving priority to the 60+ age group in the vaccination effort, due to their elevated risk levels.

The story regarding persons aged 20–39 is quite different and also more complicated. This group became eligible – in its entirety – on February 2. By that date, however, approximately one quarter of this age group *had already been vaccinated*. In part, this was due to some people under age 40 being health care professionals – a group made eligible from the start of the vaccination drive. In addition, to avoid vaccine wastage, many vaccination sites had been offering unused vaccines to any interested adult at the end of the workday.^6^ However, despite this “head start”, it took 7 weeks for 20–39 year-old age group to reach 70% first dose coverage. As of the end of March, first dose coverage had still not reached 80%, and pretty much plateaued in the low 70s.

#### Vaccine uptake by culturally defined population groups

Israel’s COVID-19 data systems track trends in positive test results, hospitalizations, mortality, and vaccinations for the entire population. The data systems also enable comparisons across population sub-groups, including ultra-Orthodox Jews, Israeli Arabs, and “the general sector” (i.e., all others). Reporting on these population sub-groups is an approximation, however, as it is based on a proxy variable reflecting the estimated size of each group in each municipality (See Appendix).

The crude comparison across population groups is further complicated by differences in age composition. On average, ultra-Orthodox Jews and Israeli Arabs have more children per family than the general sector. As a result, they are younger populations. Since older adults were made eligible for the vaccinations before younger adults, and since children under age 16 were not eligible throughout the January–March study period, between-group comparisons of crude vaccination rates per 1000 population, can be misleading, as they are not adjusted for differences in age distribution and eligibility rates.

**Figure 3:**
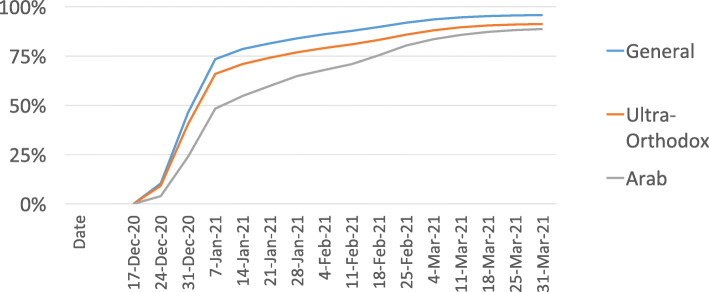
**Fig. 3** Vaccination uptake among people age 60+ (By sector and over time)

Accordingly, we focus on two particular age groups, starting with persons age 60 and over – all of whom were eligible from the start of the vaccination campaign. As can be seen in Fig. 3, at the end of December 2020, the vaccination rate among persons aged 60+ in the Arab sector (24%) was substantially lower than the rates for the general sector (46%) and the ultra-Orthodox sector (40%). As of January 28, 2021, there continued to be an absolute difference of about 20 percentage points between the Arab sector (65%) and the general sector (84%), with the rate among the ultra-Orthodox at 74%. However, by the end of March the differences had narrowed considerably: 89% in the Arab sector, 91% in the ultra-Orthodox sector, and 96% in the general sector.

**Figure 4:**
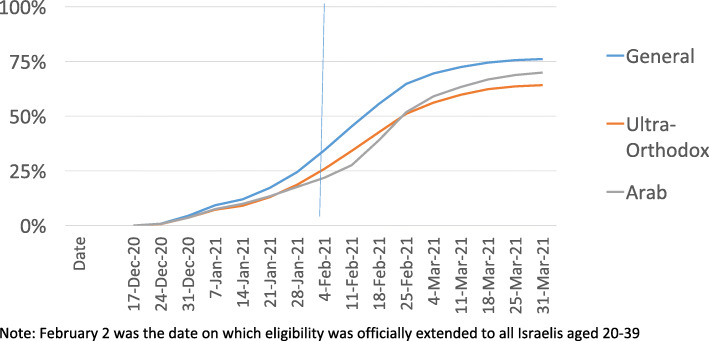
**Fig. 4** Vaccination uptake among people age 20-39 (By sector and over time)

Figure 4 presents data on vaccine coverage by population sector for the 20–39 age group. As with the 60+ age group, the general sector had the highest rate of vaccine coverage throughout the study period. Interestingly though, for the 20–39 age group, the gap between the ultra-Orthodox and general sectors increased somewhat between mid-February and the end of March, while the gap between the Arab and general sectors decreased during that period. It is also noteworthy that, by the end of March, the gaps between the sectors were similar for this age group and the 60+ age group (6 and 7 percentage points, respectively). In contrast, earlier in the study period the peak inter-sectoral gaps for the 20–39 age group were narrower than they were for the 60+ age group (17 percentage points v. 24 percentage points).

The data on which Figs. 3 and 4 are based can be found in Supplemental File 1, which focuses on uptake of first doses. Analogous data regarding the update of second doses can be found in Supplemental File 2.

Note that both crude and age-specific comparisons across the population groups are complicated by the fact that COVID-19 infection rates were higher among the ultra-Orthodox and Arab populations than among the general population, and prior infection confers a degree of immunity. Until March 3rd people who had been infected, and subsequently recovered, were not eligible for the vaccine.

Throughout the study period, vaccine uptake was markedly lower among the Bedouin in the Negev region of the country, who are a unique sub-set of Israel’s Arab population and who number approximately 270,000 (about 13% of the total Israeli Arab population and 3% of Israel’s entire population). The Bedouin population is much younger than the overall population of Israel, with only one third of the Bedouin (approximately 90 thousand people) aged 20 or over. As of the end of March, forty-four thousand Bedouin (of all ages) had received a first dose of the vaccine. This, despite significant efforts to work with local medical and religious leaders, deployment of mobile clinics, and extended hours of operation for vaccination sites.

### Factors likely to have contributed to the slowdown in the pace of vaccinations

Several inter-related factors probably contributed to the slowdown in the pace of vaccination uptake after the initial surge.

#### Impeding factors for the entire population

##### Varied proclivity within populations to adopt innovations of any type

In his classic book, “Diffusion of Innovation”, Rogers noted that with most innovations, across a wide variety of fields, products, and services, not all potential users will be equally interested in adopting the innovation immediately [15]. He describes a frequency curve of adoption and distinguishes subgroups of the entire population in terms of how rapidly they adopt an innovation: “innovators,” “early adopters,” “early majority,” “late majority,” and “laggards.”

##### Vaccine hesitancy - generic

In keeping with Rogers’ model, some people, organizations, states, and countries are quicker than others to adopt medical innovations in general, and new vaccines in particular [16, 17]. Reasons for hesitancy in adopting vaccines include concerns about vaccine safety, perceptions of low likelihood of contracting vaccine-preventable diseases (VPDs), perceived low severity of VPDs, beliefs that vaccines do not work, and overall lack of information [18]. Kumar and colleagues note that “the behaviours responsible for vaccine hesitancy can be related to confidence, convenience and complacency” [19].

Scientists often imagine that vaccine hesitancy is primarily the result of a knowledge gap: if the right knowledge is provided, the gap will be bridged, the logical conclusion will readily become apparent, and the rational decision-making process will kick in and override the emotional, illogical, misinformed position previously held. It has become increasingly clear that vaccine hesitancy cannot be fully addressed just by restating the facts. For example, in a large multi-national study, Hornsey and colleagues found anti-vaccination attitudes to be highest among those who exhibited conspiratorial thinking and those with a low tolerance for impingements on their freedoms [20]. Razai and colleagues note that higher-than-average rates of vaccine hesitancy among ethnic minorities are believed to be due in part to “the historical mistrust of government and public health bodies that runs deep in some ethnic minority groups” [21].

Interestingly, when it comes to childhood vaccinations, nation-wide coverage rates are higher among Israeli Arabs than they are among Israeli Jews [22]. Similarly, a study of routine childhood vaccinations in young children in the Jerusalem district found higher rates of completeness and timeliness for Arab children compared to Jewish children. While vaccination completeness was also reasonable among ultra-Orthodox Jews, it was lower than among Arabs and other Jews. Moreover, vaccinations among the ultra-Orthodox were less timely than among the other two groups [23].

##### Vaccine hesitancy – COVID-19

With regard to the COVID-19 vaccines, there were additional concerns related to the unprecedented speed with which there were developed [24] and that they had received emergency use authorization rather than full approval. Moreover, some of the COVID-19 vaccines, including the Pfizer-BioNTech vaccine used in Israel, were based on a highly innovative approach – the use of mRNA technology. In addition, there were claims on the part of some physicians that Israel was serving as Pfizer’s “guinea pig” for the rest of the world [25]. In addition, at that time, there was no evidence of real-world data about vaccine safety and efficacy. Israelis constituted the first population to be vaccinated *en masse*, and until publication of the early Israeli studies of the effects of mass vaccination there was a great deal of uncertainty about the extent of real-world effectiveness (i.e., beyond the highly structured context of clinical trials). Another factor that may have contributed to mistrust was that some details of the purchase agreement with Pfizer were not disclosed at the time [26].

In June 2020, when several COVID-19 vaccinations were being tested but none had yet been approved, Lazarus and colleagues carried out a large, multi-country survey of potential acceptance of those vaccines. They found that levels of trust in information from government sources was a key factor in determining potential acceptance levels [27].

##### Confusing epidemiological developments

By February 1, 2021, 34% of the total Israeli population had received a first vaccine dose, and 20% had received a second dose. Furthermore, leading Israeli researchers had begun to release findings from large-scale controlled studies indicating high levels of vaccine effectiveness [28–33]. Nonetheless, the pandemic continued to plague Israel for several weeks in February, and the number of newly diagnosed cases, hospitalizations, and deaths remained in ranges which were among their highest levels since the beginning of the pandemic. This appears to have created confusion and skepticism among the general public about whether the vaccine was as effective as it was being reported to be [34–36] and this, in turn, may have contributed to vaccine hesitancy.

In addition, several viral mutations were discovered while the vaccination campaign was underway, and several experts opined that that the vaccine was probably less effective for these variants, and might not even be effective at all. This apparently led to a feeling among some people that the benefits of getting vaccinated would be limited, since new variants are constantly evolving, and it is uncertain whether existing vaccines would be effective in addressing those variants.

Moreover, researchers and clinicians brought to public attention cases of vaccinated individuals who nonetheless contracted COVID-19. This, to some unknown extent, further eroded public confidence in the vaccine.

#### Impeding factors for specific age groups

As indicated above, in Israel, as in many other countries, young adults (aside from those at high risk due to their occupation or underlying health status) were less inclined to get vaccinated than were people over age 60. One key factor was that the health risks associated with COVID-19 are substantially lower for young adults than for older adults [37, 38]; thus, the benefits of vaccination are lower for them. In addition, younger people are more prone to be concerned about potential long-term side effects of the vaccine. One specific area of concern was over potential, albeit undocumented, long-term effects on fertility [39]. In addition, based on evidence about morbidity and mortality of COVID-19, young people in general are less concerned than older people about the personal risk posed by disease. In addition, young people are more prone to risk taking in a variety of contexts.

#### Impeding factors for culturally defined population groups

Reid and Mabhala note that, for many countries, “early studies attribute lower uptake of COVID-19 amongst ethnic minorities to the wider determinants of vaccine uptake, hesitancy or lack of vaccine confidence, including lower levels of trust and greater concerns about vaccine safety” [40]. As highlighted in recent academic and newspaper articles, some of these same factors are probably also at work in Israel [41, 42].

Highly charged concerns about fertility risks were prevalent among ultra-Orthodox Jews and Bedouin Arabs – two traditional populations that champion large families. Other challenges in promoting the vaccine among these traditional groups included their mistrust of government, their relatively limited levels of secular education, and their limited exposure to the internet, television, and mainstream media – key sources of reliable information on new scientific developments.

Moreover, anti-vax messaging was widespread on Arabic-language social media [42–45] and among ultra-Orthodox Jews. While mainstream media were used to address anti-vax messaging among the general population from early in the vaccination campaign, these media were less effective in reaching these two religious/cultural groups^7^ both because these groups are less accessible culturally and they consume less mainstream mass media than the general population.

In addition, as noted above, the proportion of the population that had already been infected and had recovered from COVID-19 was well above the Israeli average among ultra-Orthodox Jews [46] and in some Arab localities. COVID-19 recoverees were considered to have at least short-term immunity and hence were not eligible for vaccination during the first few months of the national campaign.

Another significant factor contributing to initially low vaccine uptake levels among Israeli Arabs was limited access to vaccination sites, particularly the first weeks of the vaccination campaign. This was especially problematic for Israeli Arabs living in small villages, some of which did not have their own vaccination sites. Travel to vaccination sites in larger localities is often a special challenge for Israeli Arab women with large families, due to insufficient transportation and childcare options – both public and private – combined with various cultural restrictions.

More generally, the lower vaccination rates among Arabs and the ultra-Orthodox may be related, in part, to their lower SES levels. This, despite the fact that vaccinations are available free of charge to all Israeli residents, as are most other preventive and public health services. Indeed, Caspi et al. found that in Israel there was a strong correlation at the level of the municipality between COVID-19 vaccination rates and socio-economic status (SES), and they emphasized “the need to directly target vaccination acceptance to socio-economically disadvantaged populations” [47].

The Bedouin of the Negev are a particularly low-income sub-set of the Israeli Arab population, and they are in the midst of a transition from a traditional to a modern society. Lower rates of vaccination among the Bedouin had numerous causes, including fear of vaccine side effects, mistrust of the government, limited access for Bedouin living in unrecognized villages, and complacency due to low rates of reported infection in those villages [44].

### The main steps taken to address the slowdown in vaccination uptake

The vaccine access and vaccine hesitancy challenges were addressed via a mix of messaging, incentives, and extensions to the initial vaccine delivery system. Many of the measures addressed the general population, while others targeted the most challenging subgroups. While some vaccine hesitancy remained, much progress was made.

As shown in Figs. 2 and 3 and the supplemental tables, vaccine coverage continued to increase over time for the entire population, and for the most challenging age and population sub-groups. Current rates of vaccination (overall, among young adults, and among ultra-Orthodox Jews) are all well above the receptivity rates reported in surveys that were carried out before the vaccination effort began [48]. The sections below review some of the key measures that have been taken to promote the relatively slow, but nonetheless steady, progress that has characterized Israel’s ongoing vaccination effort, following the initial rapid rollout. The overall effort was guided by the top leadership of the Ministry of Health, in consultation with a broad range of internal and external experts.

#### Promoting vaccine uptake and addressing vaccine hesitancy among the overall population

Although Israel has a legal basis for mandatory vaccinations, this has been used only twice since the founding of the state in 1948.^8^ Following the onset of the COVID-19 epidemic, a public debate arose regarding the possibility of requiring vaccination. The consensus that emerged was that the COVID-19 vaccination should not be compulsory, but that positive and negative incentives could be created to encourage vaccination uptake (including incentives that would require new legislation).

Several of the most significant steps taken by Israel that encouraged the population to get vaccinated were taken, or were at least initiated, before or during the initial phase of the vaccination program. These included holding off the program until the Pfizer vaccine had been approved by the FDA for emergency use; making vaccines readily available to all Israelis at no cost (“making the right choice the easy choice”); and showcasing in the mainstream media both the large numbers of Israelis presenting at vaccination sites and the enthusiasm of the newly vaccinated [1].

Levin-Zamir et al. have observed that the success of Israel’s vaccination program depended not just on nationwide accessibility of vaccine [49]. Other important factors were “building public trust through an integrated and familiar health system, a familiar technology (vaccine), transparency regarding vaccine safety information, culturally appropriate messages in digital and offline media, acknowledging diverse health literacy needs, and active participation and role-modeling by political/religious opinion leaders.”

##### Promoting uptake through public education, communication, and messaging

As the vaccination program proceeded, it evolved to address the apparent issues associated with the slowdown in the pace of vaccinations. New elements included a coordinated and simultaneous response by senior officials of the Ministry of Health, the health plans, various experts and public figures, to anti-vax messaging using mass media, social media and other channels; publication of daily updates on vaccination coverage by age group and locality, and the publication of a steady stream of findings from Israeli studies that demonstrated that the real world effectiveness of the vaccine beyond the clinical trial setting^9^ [28–30]. Political and health care leaders also communicated a vision of how widespread uptake would enable a return to normalcy within the foreseeable future.

Israeli public health experts played two very important roles in addressing the confusion created in early February by continued high infection rates despite growing levels of vaccination coverage. First, they immediately provided plausible explanations for the paradox. Second, they subsequently provided early, real world evidence that the vaccination program was having a demonstrable, population-level, positive impact in Israel. The explanations for the paradox included the arrival in Israel of a new, highly infectious strain (B.1.1.7, the UK variant),^10^ the necessity of a second vaccine dose to reach maximal immunity, and the lag time between vaccination and the full impact on immunity [34–36].

The population-level evidence enabled researchers to take advantage of several opportunities to analyze data from natural experiments [50, 51]. These included insightful cross-sectional comparisons of morbidity indicators across municipalities with different levels of vaccination coverage, and comparisons of trends across age groups with different levels of vaccination coverage (related to differences in the dates at which they became eligible). For example, they demonstrated that among persons aged 60+, hospitalizations declined sooner than they did for other age groups. Importantly, and with great benefit to Israel’s vaccination effort, many researchers did not delay sharing their findings until the journals they had submitted to for publication completed the peer review processes. Instead, key findings were shared with the public several weeks, or even months, before the full studies were published in scientific journals.^11^

##### Promoting uptake through outreach

In the first month of the rollout, almost all vaccinations of Israelis residing in the community, aside from hospital employees, were administered by the health plans.^12^ Even as the vaccination effort evolved, the health plans continued to be the predominant agents for administering vaccinations. However, their work was supplemented by municipalities, the IDF home front command, and other organizations that were well positioned to organize delivery sites and events that were tailored to particular population segments. This work was typically done in cooperation with the health plans and/or Magen David Adom (MDA - Israel’s national emergency medical service), who handled the actual administration of the vaccines as well as other aspects of the effort requiring vaccine-related expertise such as cold chain preservation. For example, mobile vaccination units serving members of all health plans, and operated by MDA or commercial vendors, were placed on university campuses, commercial areas, and in villages where residents had relatively limited access to fixed vaccination sites.

Special outreach efforts were particularly important for the infirm, mostly elderly, population. The MDA was the central player in bringing vaccines to all nursing homes and geriatric facilities. The health plans, together with the local authorities, transported homebound patients by car or ambulance to vaccination centers. Later on, when an appropriate vaccine transport method was developed, health plan personnel brought the vaccines to the homes of homebound patients, and vaccinated them there.

Steps were also taken to vaccinate several special populations who do not have Israeli residency (and hence are not included in the official vaccination coverage statistics). These included foreign workers,^13^ (including those in the country without the necessary documentation), undocumented immigrants,^14^ and Palestinians working in Israel, who were vaccinated at centers set up at entry points into Israel.

##### Promoting uptake through strategic linkages and incentives

The early stages of the vaccination program proceeded largely on a separate track from other efforts to combat the spread of the pandemic and gradually return the economy and the society to routine activity. As more and more of the population became vaccinated, these efforts became increasingly intertwined.

For example, starting on February 11, the government decided that the extent of school reopening would vary by municipality or neighborhood.^15^ Initially, this authorization to open schools was dependent solely on the rate of disease transmission in that municipality or neighborhood. Subsequently, and starting on February 22, the municipality’s vaccination uptake level was also factored directly into decisions about reopening schools.^16^ The effect was to create a municipal-level or neighborhood-level incentive to promote vaccinations.

Another example relates to incentives at the individual level. On February 18, the government initiated the Green Pass program. A Green Pass was issued to individuals who had either recovered from COVID-19 or who were fully vaccinated against COVID-19 (i.e., a week after receipt of a second dose). Initially, the Green Passes exempted from quarantine people who had been exposed to established cases. They also entitled their holders to “use gyms and pools, attend sporting and culture events, and stay at hotels” from which non-pass holders were excluded [13, 63]. On March 19, entry to indoor events and sporting matches was broadened from Green Pass holders to include people who had recently had a negative rapid COVID-19 test.^17^

One of the primary objectives of the Green Pass was to facilitate return to routine social activity while controlling the spread of infection. It also had the effect of creating an individual-level incentive to get vaccinated [64]. Many individuals who had previously held off on getting vaccinated, now had additional motivation to get vaccinated - the opportunity to partake in a growing range of cultural, social, religious, and other activities – without the need for frequent testing.

The Green Pass can be considered both as a “carrot” to encourage people to get vaccinated and a “stick” to pressure people who are disinclined to get vaccinated. From a legal and ethical point of view, this program was considered controversial by some.

In the prevalent Western worldview, the Green Pass program may provoke allegations of violation of ethical limits to intrusion into the autonomy of the individual [65, 66]. For example, in the U.S. there is strong public and policy opposition to requiring presentation of proof of vaccination against COVID 19.^18^ The WHO, among others, raised concerns that such a document would create ‘two types of citizen’: the vaccinated and the non-vaccinated. This seems especially unfair to those in the many countries where it is still so difficult to access vaccines. Among western nations, there is a broad consensus that programs of this sort should be considered justified only in situations where they are needed to contain major threats to public health and should be limited to the duration of the threat.

Planning for the Green Pass program began long before its February 18 launch, and these planning efforts were widely publicized in the media. It is quite likely that this helped encourage vaccine uptake even before the program was implemented; many individuals were likely eager to position themselves to be able to take advantage of future opportunities, even though uncertainty remained about exactly when and how the Green Pass would be enacted.

Some other incentives and penalties that Israel considered are also complicated from a legal and ethical point of view, and there is still no final decision in the courts on their legal status. For example, a law has been enacted that allows the Ministry of Health to provide local authorities with information about the vaccine status of their residents, but Israel’s Supreme Court is currently undertaking a judicial review of the validity of this law. Some local authorities have banned people who are not vaccinated from working directly with children, and there is a lower court decision endorsing this, but that is not considered a binding precedent and is open to appeal. There were also strong differences of opinion about the appropriateness of efforts to promote vaccination that were targeted at teachers and other specific employee groups [26].

In addition to the incentives introduced by government, some private companies have offered special one-time payments to their employees who get vaccinated.

#### Special steps taken to promote vaccine uptake and address vaccine hesitancy among young adults

By March 31, 73% of 20–29 year olds had been vaccinated, despite an initially slow uptake and despite the initial hesitancy prevalent among this age group, as noted above. This section reviews some of the main steps that contributed to the increase in uptake.

One of the main efforts undertaken to promote uptake and address vaccine hesitancy among young adults consisted of targeted messaging that highlighted expert opinion on vaccine safety. This included messages about vaccine safety in general, messages about the lack of a scientific basis to claims about long-term risks or about risks to fertility, and messages about the limited risks specific to pregnant women [68–70].

To reduce hesitancy among young women concerned with effects of the vaccine on fertility, on their infants, and on other issues, the Ministry of Health opened, in March 2021, a special center and phone line at well-baby clinics with nurses trained to answer to those specific questions [71]. News items about several pregnant women (and their fetuses) dying of COVID-19 [68, 69, 72, 73] apparently also contributed to an increase in vaccine uptake among pregnant women [74].

In addition, Israel invested significant efforts in communicating the message that, while the risk of mortality and severe morbidity from COVID-19 is particularly great among the elderly, the disease also has substantial risks for all age groups, including young adults. The messaging included systematic data and case reports of young people who had died or become seriously ill after contracting COVID-19. Over time, evidence accumulated that vaccinations of pregnant women were safe and did not have reproduction-related side effects. This information was added to the messaging.

In keeping with the notion of making the healthy choices the easiest choices, Israel also launched numerous vaccination events in places where young people gather, such as universities and downtown nightlife districts. This not only increased convenience at the individual level; it also promoted a herd effect and a perception that getting vaccinated was something that young people were happy to do.

Finally, it is likely that the Green Pass program, which was initiated for the entire population, may have been particularly influential among young adults, who are major consumers of the cultural and sporting events that became accessible through vaccination.

#### Special steps taken to promote vaccine uptake and address vaccine hesitancy among culturally defined population groups

The uptake among ultra-Orthodox Jews and among Israeli Arabs was slower than among the general population, and this was true for all age groups. Nonetheless, by March 31, 74% of ultra-Orthodox Jews over age 20 had been vaccinated, as had 75% of Israeli Arabs in that age group. Moreover, among people aged 60 and over, 91% of ultra-Orthodox Jews and 89% of Israeli Arabs had been vaccinated by March 31. This section reviews some of the main steps that contributed to the progress that was made in vaccinating these two cultural minorities.

##### The establishment of focused task forces

In August 202, the management of Israel’s national program for addressing COVID-19 was assigned to Magen Israel, a new organizational unit with Ministry of Health leadership. Magen Israel then established a special task force charged with focusing on the population of ultra-Orthodox Jews and another special task force focused on the Israeli Arab population. These task forces were established in consultation with leaders of the relevant communities and included professionals from within those communities. Subsequently, a third task force was established to address the needs on the “general” population – Israelis who are neither Arabs nor ultra-Orthodox Jews, but here we focus on the other two task forces.

Throughout much of 2020, the main responsibilities of the task forces were to disrupt the chain of transmission by encouraging compliance with COVID-19 restrictions: physical distancing, the wearing of face masks, the appropriate use of testing, and adherence to isolation and quarantine directives. To that end, each task force developed close working relationships with leaders of their target population, analyzed the group-specific barriers to desired behaviors, and developed tailored strategies for promoting desired behaviors. Their work reflected the understanding that neither the Israeli Arab nor the ultra-Orthodox communities are monolithic, with further tailoring needed for specific localities and subgroups, such as the Bedouin and Hassidic communities.

Toward the end of 2020, these task forces were also charged with promoting vaccine uptake. They did so through four main strategies: analysis of the reasons for slow vaccine uptake, partnership with community leaders, tailored messaging, and easing access to vaccination sites.

Significantly, the work of the task forces in promoting vaccine development benefited from their earlier work on other aspects of pandemic control, and lessons learned from the successes and failures of that earlier work. For example, when COVID-19 testing sites were first introduced, it took the Israeli health care system several months to realize that, while simply announcing the availability of COVID-19 tests worked well for the general population, more active outreach was needed for the Israeli Arab population [75]. The Arab task force learned from that experience, so that when the vaccine became available, it quickly recognized the need for tailored, active outreach.

##### Analysis of the reasons for slow vaccine uptake

Within the first weeks of the vaccination campaign, it became apparent to the professionals working at the Arab population task force that vaccine uptake among Israeli Arabs was substantially slower than in the general population. The task force quickly undertook an analysis of the causes of the relatively low uptake and concluded that it was multi-factorial, and included barriers to access, insufficient appreciation of vaccination benefits, and exaggerated fears of vaccine risks. The task force’s earlier work for and with the Israeli Arab population helped it complete this analysis quickly and effectively, as it had already built a team of dedicated experts and channels of communication with leaders of the Arab population. This, in turn, helped it to promptly develop and implement appropriate interventions.

The ultra-Orthodox task force undertook a similar analysis regarding its target population and acted accordingly.

##### Partnering with community leaders

Even before the vaccine had been approved for use by the Ministry of Health, the ultra-Orthodox task force solicited letters of support from rabbinic leaders encouraging vaccination, and rabbinic and communal leaders were recruited to encourage compliance throughout the vaccination campaign. In addition, senior physicians who had close ties to ultra-Orthodox Jewish communities (and who were highly respected by them) were enlisted to counter concerns about vaccine safety, and family physicians were mobilized to do vaccine promotion outreach among their patients. The task force brought together trusted physicians and rabbinic leaders to address the concern about possible side-effects on fertility, which were widespread particularly among young ultra-Orthodox women. These outreach efforts on the part of physicians built on the mainstream ultra-Orthodox community’s long-standing acceptance of modern medicine and its respect for physicians. They were particularly important in dispelling concerns about fertility risks.

Many ultra-Orthodox Jews live in homogenous municipalities. Their mayors were successfully included in vaccination campaigns – both to call upon their residents to get vaccinated, and to provide practical logistical support.

Similarly, many Muslim religious leaders, Arab physicians, and Arab soccer stars responded to requests from Magen Israel that they actively and publicly encourage Israeli Arabs to get vaccinated. Mayors and other leaders of Arab municipalities also spoke out in favor of vaccination and took concrete steps to make it easier for residents of their municipalities to reach vaccination sites. Moreover, Magen Israel learned to respond promptly and effectively to requests from municipal leaders to cooperate with them on promoting vaccination uptake. The Israel Defense Forces’ Home Front Command also contributed to these cooperative efforts.

##### Tailored messaging

While the uptake of vaccines among ultra-Orthodox Jews was slower than among the general population, it did not face substantial resistance. This contrasts sharply with the resistance by some sects of ultra-Orthodox Jews to physical distancing, school closures, and rules limiting the sizes of weddings, funerals, and prayer gatherings – particularly during the first wave of the pandemic. This general openness to vaccinations made it possible for the agencies and professionals involved in promoting vaccinations to rely almost exclusively on encouragement and facilitation, as opposed to more confrontational approaches.

Messaging efforts increasingly made use of communication media specific to the ultra-Orthodox Jewish population, including magazines, newsletters, recorded phone messages, and billboard posters. These were written using messages, wording, and language (i.e., Yiddish in addition to Hebrew) tailored to the community. For example, many of the communications cited relevant Biblical verses, such as “Take good care of your life” (Deuteronomy 4:15) and “Do not stand idly by when your colleague is at risk” (Leviticus 19:16).

There was some anti-vax messaging and other forms of misinformation in the ultra-Orthodox communities, some of it originating with charismatic rabbis, particularly during the early weeks of the vaccination campaign. These messages were spread largely through flyers placed in mailboxes and posters pasted on billboards – both of which are typic and effective ways of spreading messages in ultra-Orthodox communities. However, the anti-vax messaging was relatively limited in scope, duration, and influence, in part due to effective responses on the part of local authorities and the IDF’s home front command.

Anti-vax messaging and misinformation was more widespread in the Arabic-language social media, which significantly exaggerated vaccine risks (particularly regarding fertility) and which promoted distrust of government. The vaccine promoting messaging – emphasizing vaccine efficacy and safety - was done via Arabic-language traditional media and social media, and via individual outreach by the health plans and Arab physicians. Muslim religious leaders and various voluntary organizations (such as the Association of Arab Physicians in the Negev) also played an important role in the messaging effort.

The messages themselves were also tailored to the Israeli Arab population and culture. For example, one of the health plans broadcast a clip of a woman wearing a traditional headscarf saying that it is permissible to get vaccinated during the Ramadan fast. In addition, some of the exhortations to get vaccinated cited traditional Arab proverbs on the importance of honoring the elders of the community.

##### Easing access to vaccination sites

In keeping with Israel’s overall approach to vaccine administration, the health plans were the main organizations charged with vaccine administration in ultra-Orthodox and Arab cities and towns, with each health plan responsible for vaccinating its own members.

In the ultra-Orthodox sector, this was supplemented by the work of MDA that established pop-up vaccination sites for members of all health plans in ultra-Orthodox *yeshivas* (centers for advanced Jewish studies) and other key locations in ultra-Orthodox cities and neighborhoods. Comfort foods of special resonance for ultra-Orthodox Jews, such as *cholent,*^19^ was served free at some of these pop-up sites to encourage people to come and get vaccinated. The provision of culturally appropriate comfort foods may have served to both attract more people to the events in which they were served, and to broadcast a more generic message that the organizations mounting the vaccination efforts cared about cultural minorities.

One of the barriers to vaccination among some of the ultra-Orthodox was distance from, and physical access to, vaccination sites, due to low car ownership rates and a high reliance on sparse public transportation. For many, this barrier also included childcare issues, due to the high prevalence of large families with small children.

In many ultra-Orthodox localities and neighborhoods, early on in the pandemic, the task force appointed a coronavirus coordinator for each apartment building. At first, the coordinator’s main responsibility was to promote social distancing. These same coordinators were now mobilized to encourage vaccination and to facilitate transportation and child-care solutions, making it easier for individuals to reach the vaccination sites.

Mobile units were also an important component of the outreach effort to Israeli Arabs; initially these were operated by the MDA, and subsequently by a commercial vendor. These were particularly important for improving access to Arabs living in small villages that did not have clinics of sufficient size and technical sophistication to accommodate the demanding cold storage requirements of the Pfizer vaccine.

## Discussion

As vaccine rollouts progress in countries around the world, and a growing percentage of their populations are vaccinated, new challenges are emerging that were not prominent at earlier stages. Israel continues to be ahead of most countries in terms of both first and second dose coverage. As such, it has encountered several challenges earlier than many other countries.

### Summary of the main challenges facing vaccine uptake

The main challenge Israel faced, after the initial sprint of its vaccination campaign, was vaccine hesitancy, while pockets of limited geographic access constituted an additional challenge. Israel had to address a non-negligible amount of vaccine hesitancy in its general population, along with more intense pockets of vaccine hesitancy among young adults, and ultra-Orthodox Jews and Israeli Arabs – religious/cultural minorities which tend to be more traditional. For a segment of Israeli Arabs, difficulties in reaching vaccination sites also slowed the pace of vaccination. For the general population, vaccine hesitancy was abetted by the confusion created by a continued high rate of infection in early February, despite broad vaccination coverage.

### Success in vaccine procurement and administration is not enough to ensure adequate vaccine uptake

The challenges to vaccine uptake were addressed via a mix of messaging, incentives, and extensions to the initial vaccine delivery system. Many of the measures addressed the general population, while others were targeted at the most challenging subgroups. Some of these measures may be adaptable to other countries if, and when, they encounter a substantial amount of vaccine hesitancy and/or geographic access challenges.

Significantly, the capacities and strategies that enabled Israel to rapidly procure, distribute and administer the vaccines (listed in Table 1, as items 1–11) were not sufficient for ensuring sufficient uptake of the vaccine. As indicated in the first part of Table 6, several of those factors also contributed to vaccination uptake. However, as suggested by the observed slowdown in vaccine uptake, those factors alone would probably not have been sufficient to enable Israel to promptly reach the levels of coverage needed to restore normal function to society and the economy [12].

**Table 6:** Capacities and strategies that facilitated vaccine uptake

**A. Capacities and strategies that were also vital to procurement and distribution**	
1. Israel’s experience in, and infrastructure for, planning and implementing prompt responses to large-scale national emergencies	
2. The tradition of effective cooperation between government, health plans, hospitals, and emergency care providers – particularly during national emergencies – and the frameworks for facilitating that cooperation 3. The organizational, IT and logistic capacities of Israel’s community-based healthcare providers	
4. The existence of well-functioning frameworks for making decisions about vaccinations and support tools for assisting in the implementation of vaccination campaigns	
**B. Additional capacities and strategies that were not vital to procurement and distribution**	
5. An ability to track vaccination uptake by age, population group, and locality	
6. A willingness to adopt new vaccine distribution mechanisms and partners	
7. Well-tailored outreach efforts to encourage the population to sign up for vaccinations	
8. A willingness and capacity to address head-on the unique needs of cultural minorities	
9. A capacity for mounting effective information campaigns	
10. The judicious use of incentives	
11. Patience and perseverance	

Fortunately, as indicated in the second part of Table 6, Israel was able to supplement the capacities and strategies it used to ensure rapid procurement and distribution with additional capacities and strategies that enabled it to promote sustained vaccination uptake. They included an ability to track vaccination uptake by age, population group, and locality; a willingness to address head-on the unique needs of cultural minorities and a capacity to mobilize accordingly; a willingness to adopt new vaccine distribution mechanisms and partners; the judicious use of incentives; patience and perseverance; and a capacity for mounting effective information campaigns.

Interestingly, being a small country was helpful to Israel in the initial phase, making it easier to promptly procure an adequate supply of vaccines and distribute them across the county. However, even in a relatively small country with fewer than 10 million people, the population is not homogeneous, which should be accounted for in designing and implementing an immunization program intended for rapid uptake in the entire population.

### Recognizing and addressing the communal aspect of vaccine uptake

The establishment of special task forces to work closely with the Israeli Arab and ultra-Orthodox Jewish communities on pandemic control probably contributed significantly to the closing of the initial gaps in vaccination uptake. These two large population groups in Israel had relatively high concentrations of residents reluctant to take the COVID-19 vaccine. Despite the vast religious and cultural differences that separate these two groups, they do share at least two key attributes. One is a deep-rooted suspicion of government. While both these population groups traditionally share a strong respect for authority, it is primarily for the authority of religious or social community leaders and substantially less so for the authority of secular government. The second has been the maintenance of a strong communal identity and a distinct set of cultural, social and political institutions. For example, each of these groups operates its own, state-funded educational system as well as several political parties with representation in the Knesset. Thus, to promote vaccination uptake among Israel Arabs and ultra-Orthodox Jews, it was important to engage them at the communal/sectoral level, in addition to reaching out to them as Israeli citizens and as individuals.^20^

### Study limitations and suggestions for further study

While the analysis of vaccine uptake over time was based on data, the analysis of barriers to uptake and the steps taken to overcome those barriers is based largely on a limited number of interviews with key informants and a review of publicly available sources. More in-depth analyses based on surveys or archival analysis could result in the identification of additional factors.

The present study also did not seek to tease out the effects of the specific interventions. Doing so is a challenging task, beyond the scope of this study, due to the simultaneity of many of the interventions and likely time lags in their effects. However, the identification of such effects could be a very useful input into the design of policy responses to future large-scale emergencies, and hopefully this challenge will be undertaken by scholars in Israel and abroad.

### Opportunities for cross-national learning

Vaccine hesitancy is now considered the major hurdle to controlling the pandemic in many countries. Moreover, many vaccine hesitant people around the world express positions heard in Israel, namely that COVID-19 vaccines were brought to market too quickly for meaningful analysis of potential side effects; that they prefer to “wait and see”; and a non-evidence based gut risk-benefit analysis, the conclusion of which is that infection with coronavirus poses less personal risk than getting vaccinated, and misinformation regarding concerns with short and long term side effects. Israel and many other countries also have various communities that maintain alternative lifestyles, and who refuse vaccination based on non-scientific belief systems. The pockets of relatively widespread vaccine hesitancy that were found in Israel among young adults and cultural minorities have their parallels in other countries as well. Accordingly, Israel and other countries can probably glean useful ideas from one another’s efforts to address vaccine hesitancy. For example, the ways that Israel mobilized to promote vaccine uptake among its cultural minorities, might provide ideas for other countries which could then be adapted to each country’s unique social and political context.

### The Israeli experience - a reminder of the need to meld optimism with caution

The Israeli experience indicates that it can be difficult to sustain a fast pace of vaccination for extended periods. It may get easier over time to increase the capacity to vaccinate; vaccine production ramps up and kinks in the delivery system get worked out. However, the passage of time is not always as favorable to vaccine uptake. In Israel’s experience, after the early adopters had been vaccinated, the pace of vaccination uptake slowed, and the public health system had to work hard and resourcefully to increase population coverage from 40 to 60% and beyond.

While the Israeli experience provides a reminder of the need to be cautious in forecasting the pace of vaccinations, it can also be a source for optimism and practical ideas. With hard work, creativity, and patience, Israel is succeeding in encouraging vaccination uptake among more and more of its citizens who were not early and eager adopters of the COVID-19 vaccine.

In addition, the Israeli experience suggests that life can increasingly go back to normal even before achieving full herd immunity or even a 70% vaccination coverage rate. By mid-April, with vaccination coverage still at around 60%, and with no vaccines approved as yet for children, Israel was still able to open up much of its society and economy without this resulting in a major upsurge in the pandemic at that time.

In recent months, the Israeli government has continued to be vigilant, realizing that there is a portion of the population that is still unvaccinated and that, despite stringent efforts to control entry of potentially infected persons, it is likely to be impossible to ensure that there is no importation of new variants of SARS-CoV-2. As of the time of this writing (early July 2021), Israel is facing another increase in COVID-19 cases, due mainly to the spread of the delta variant among children. While previous vaccination rates were high enough to substantially diminish viral spread at the population level, in mid-2021 the delta variant’s rapid spread is posing a new challenge to the Israeli healthcare system and Israeli society. Another proactive push to vaccinate is underway and it is needed particularly among more than 200,000 Israelis above age 50 who are not yet vaccinated, and among the recently eligible 12–15 age group. In the first 3 weeks of the campaign to vaccination 12–15 year olds, uptake among the Arab and Ultra-orthodox communities were below the national average (as had been the case for other age groups). Some of the remedial actions undertaken for older age groups in these communities, as described in this paper, should be considered for 12–15 year olds, and adapted accordingly.

Overall, the hope is that more and more countries will be able to vaccinate a high percentage of their populations with the most effective vaccines they can obtain, understand barriers to achieving 100% vaccination, and address them. In doing so, they are likely to contribute additional lessons to conducting effective national immunization programs. More importantly, they will be lessening the impact of COVID-19 on their population, helping to restore more normal conditions, and contributing to global control of the COVID-19 pandemic.

### Supplementary Information

**Additional file 1: Supplemental file 1. **– tables with data on uptake of first dose

**Additional file 2: Supplemental file 2.** – tables with data on uptake of second dose.

## Data Availability

Not applicable.

## Appendix

### The methodology for computing age and sector specific vaccination rates

The Ministry of Health publishes daily updates on the number of vaccinated persons both nationally and by locality, but does so without differentiation among sectors. For our manuscript, estimates of the country-wide number of vaccinated persons in a particular sector (Arab, ultra-Orthodox, or general) were calculated as aggregates of the number of vaccinated persons in that sector in each of Israel’s localities.

Sector-specific estimates of the number of vaccinated persons in a particular locality is relatively simple in the case of localities which are comprised almost exclusively of individuals from one sector, and most Arabs and ultra-Orthodox Israelis live in such localities. However, many others live in mixed localities, for which the calculations are more complicated and more susceptible to bias and other types of estimation errors. To estimate what share of a mixed locality’s residents (and what share of the locality’s vaccinated residents) come from a particular sector, we made use of a methodology similar to one developed and used by the Central Bureau of Statistics for other purposes [76]. The proportion of residents and vaccinated residents who are Arab is estimated to be the same as the share of residents who voted for predominantly Arab parties in the most recent national election, and a similar estimation technique is applied to develop estimates for the ultra-Orthodox population. The estimates for the general population are calculated as residuals (General population = Total population – Arab population – ultra-Orthodox population).

While this approach is recognized to be quite effective for estimating the number of residents of a locality from each sector, it is potentially problematic for estimating the number of vaccinated residents from each sector, as it implicitly assumes that within each locality the vaccination rates are equal across sectors.

Accordingly, as a sensitivity analysis we also analyzed trends over time in sector-specific vaccination rates, after excluding localities in which none of the three sectors accounted for 80% or more of the population. The resulting estimates of vaccination uptake were slightly different from those of the main analysis; as expected, they were slightly higher for the general sector and slightly lower in the Arab and ultra-Orthodox sectors. Nonetheless, the overall patterns of inter-sectoral gaps and how they evolved over time were very similar to what was found in the main analysis.

Thus, the sensitivity analysis provides reassurance regarding the main story line presented in this article. In the future, it will be possible to develop more precise estimates of sector-specific vaccination uptake if, and when, the data on vaccinations are made available at the level of the statistical area (which tend to be more homogenous than localities).

## Abbreviations

MDA: Magen David Adom
SES: Socio-economic status
IDF: Israel Defense Forces

## Footnotes

1 While this article focuses on first vaccine doses, there are several important points to make about administering second doses in Israel, as follows: 1. Israel adopted a two dose regimen for the Pfizer vaccination; 2. It invested substantial energy in promoting second doses as being vital to ensuring maximal protection; 3. Over 90% of Israelis who received a first dose, have subsequently received a second dose; 4. Israel’s success in second dose coverage is due in part to its health plans’ tracking systems - which enabled them to store and retrieve information on which of their members had received which doses - and their communication system for reaching out effectively to members who had not yet received a second dose.

2 Unlike most other vaccines available in early 2021, the Pfizer vaccine - on which Israel relied - was authorized for use for persons age 16 and over.

3 The available comparative data relate to the percentage of the population that is under age 15, and not the percentage under age 16. As of 2018, the percentage under age 15 was 28% for Israel and 18% for the OECD average [10]

4 The decision to vaccinate 16–18 year olds before vaccinating most 18–40 year olds was taken, in large part, to help high school juniors and seniors successfully complete their matriculation exams. A related factor may have been the recognition that those age groups with particularly high COVID-19 mortality rates had already been covered.

5 The vaccines were initially administered primarily in designated large complexes that have been either established or converted for the purpose of the vaccination campaign, led by the control center of the MoH and with the assistance of the Home Front Command of the army, and in cooperation with the local authorities. These operated in long shifts and weekends. During a second stage, starting in January 2021, less than a month after the vaccination campaign has started, the health plans moved most of the vaccination sites from big complexes to their primary care clinics spread in the community, that work normal shifts, and which are closed during weekends [13]. Significantly, even in late December and early January, when the large complexes were the dominant delivery sites, fewer vaccinations were administered on Fridays and Saturdays than during weekdays; apparently this was as a result of lesser demand during weekends.

6 This generated media interest and public excitement, which may have pre-empted vaccine hesitancy among younger persons for when they subsequently became eligible as a group.

7 Both the Arab and ultra-Orthodox populations prefer sectoral newspapers and radio stations, as they find them more compatible in terms of language, content, and culture.

8 The Public Health Ordinance of 1940 (Article 19) allows for forced vaccination of the population in Israel in situations of an epidemic that immediately and clearly endangers the whole of society. That authorization has only been used twice: in 1949 during an outbreak of smallpox and in the early 1990s during an outbreak of measles.

9 In mid-February Israel’s two largest health plans released preliminary findings regarding the health status of members who had been fully vaccinated. Clalit (which has approximately 4 million members) reported on a study comparing 600,000 fully vaccinated members with a similarly sized, matched group of unvaccinated members. The study found that the vaccine was 94% effective in preventing symptomatic COVID-19, and 92% effective in averting serious cases of the disease [32]. Maccabi (which has approximately 2 million members) reported that, among its 600,000 fully vaccinated members, only 1% had contracted COVID-19, and none had died [28].

10 While the Pfizer vaccine is effective in preventing the spread of the UK variant (B.1.1.7), that variant’s highly infectious nature facilitated its spread among those who had not been vaccinated.

11 In recent months, several of these studies have been published in some of the world’s most prestigious journals [9, 50–55] . Pre-prints were published in MedRxiv [47, 56, 57], SSRN [58, 59], Twitter [60], and other platforms. The media also played an important role in communicating experts’ explanations and findings with the general public.

12 Hospitals vaccinated their own employees, while residents of long-term care facilities were vaccinated by Magen David Adom – Israel’s national emergency services organization. Soldiers were vaccinated by the IDF Medical Corps.

13 In Israel, all employers of legal/documented foreign workers are required to purchase private insurance for their workers. This made the workers eligible for vaccinations through the health plans with which the private insures contract. Foreign workers providing long-term care services also had the option of being vaccinated alongside their frail employers by their employers’ health plans at an early stage of the vaccination campaign [61].

14 A large numbers of Israel’s homeless and undocumented migrants reside in Tel Aviv. That municipality (and not the health plans) took on the responsibility of vaccinating this population. Together with an NGO and the municipal hospital, the municipality opened a vaccination center to provide both doses free of charge [62].

15 In Israel this was called “the traffic light system”, with each municipality assigned a green, yellow, orange or red rating, with implications for the extent to which they could open schools.

16 Interestingly, government did not acquiesce to calls from parents to forbid in-person instruction by non-vaccinated teachers [26].

17 There is a concern that this ruling, along with the growth of opportunities for rapid and inexpensive testing, may reduce the incentive to get vaccinated that was created by the Green Pass program.

18 However, enrollment in kindergartens is typically dependent on proof of receipt of vaccinations against childhood infectious diseases [67].

19 This was a cultural adaptation of the custom of ultra-Orthodox yeshiva students to go out on Thursday evenings and eat *cholent*, after a week of intensive study. *Cholent* is a stew typically consisting of meat, potatoes, onions and other vegetables.

20 As noted above, inadequate access to reliable information about the vaccine safety and effectiveness, and misinformation was greater among the members of these communities. In addition, individuals in both of these communities tend to have large families, low SES, and poorer access to health care. Therefore, stronger efforts were required to improve access to the vaccine, including both physical access (distance and transportation solutions), and cultural access (e.g. solutions for child care while absent getting the jab, or solutions for traditional Muslim women to leave their homes).
