# Supplementary material for: Addressing vaccine hesitancy and access barriers to achieve persistent progress in Israel’s COVID-19 vaccination program
Source: Isr J Health Policy Res. 2021 Aug 2;10:43. doi: 10.1186/s13584-021-00481-x (PMC8326649; doi:10.1186/s13584-021-00481-x)
Supplement: Supplementary file 1 — Additional file 1: Supplemental file 1. – tables with data on uptake of first dose [file 13584_2021_481_MOESM1_ESM.docx]

**Supplemental file 1 – tables with data on uptake of first dose**

| **Table 1** |  |  |  |  |  |
| --- | --- | --- | --- | --- | --- |
| **Cumulative percent vaccinated with first dose, by date and age group** | | | | | |
|  | |  |  |  |  |
|  |  |  |  |  |  |
| **Date** | **Age 20+** | **Age 20-39** | **Age 40-59** | **Age 60+** |  |
|  |  |  |  |  |  |
| 24-Dec-20 | 3% | 1% | 2% | 10% |  |
| 31-Dec-20 | 16% | 4% | 10% | 44% |  |
| 7-Jan-21 | 29% | 9% | 23% | 70% |  |
| 14-Jan-21 | 34% | 11% | 31% | 76% |  |
| 21-Jan-21 | 41% | 16% | 43% | 79% |  |
| 28-Jan-21 | 47% | 22% | 52% | 81% |  |
| 4-Feb-21 | 54% | 31% | 59% | 84% |  |
| 11-Feb-21 | 60% | 40% | 63% | 85% |  |
| 18-Feb-21 | 66% | 51% | 69% | 88% |  |
| 25-Feb-21 | 73% | 60% | 74% | 90% |  |
| 4-Mar-21 | 77% | 66% | 78% | 92% |  |
| 11-Mar-21 | 79% | 69% | 80% | 93% |  |
| 18-Mar-21 | 81% | 71% | 81% | 94% |  |
| 25-Mar-21 | 81% | 72% | 82% | 94% |  |
| 31-Mar-21 | 82% | 73% | 82% | 95% |  |

| **Table 2** |  |  |  |  |
| --- | --- | --- | --- | --- |
| **Cumulative percent vaccinated with first dose, by date and sector** | | | |  |
| **Persons age 60+** | |  |  |  |
|  |  |  |  |  |
|  | **Total - All** | **Arab** | **Ultra-Orthodox** | **General** |
| **Date** | **Sectors** | **Sector** | **Sector** | **Sector** |
|  |  |  |  |  |
| 17-Dec-20 | 0% | 0% | 0% | 0% |
| 24-Dec-20 | 10% | 4% | 9% | 10% |
| 31-Dec-20 | 44% | 24% | 40% | 46% |
| 7-Jan-21 | 70% | 48% | 66% | 73% |
| 14-Jan-21 | 76% | 55% | 71% | 79% |
| 21-Jan-21 | 79% | 60% | 74% | 81% |
| 28-Jan-21 | 81% | 65% | 77% | 84% |
| 4-Feb-21 | 84% | 68% | 79% | 86% |
| 11-Feb-21 | 85% | 71% | 81% | 88% |
| 18-Feb-21 | 88% | 75% | 83% | 90% |
| 25-Feb-21 | 90% | 80% | 86% | 92% |
| 4-Mar-21 | 92% | 84% | 88% | 94% |
| 11-Mar-21 | 93% | 86% | 90% | 95% |
| 18-Mar-21 | 94% | 87% | 91% | 95% |
| 25-Mar-21 | 94% | 88% | 91% | 96% |
| 31-Mar-21 | 95% | 89% | 91% | 96% |

| **Table 3** |  |  |  |  |
| --- | --- | --- | --- | --- |
| **Cumulative percent vaccinated with first dose, by date and sector** | | | |  |
| **Persons age 20-39** | |  |  |  |
|  |  |  |  |  |
|  | **Total - All** | **Arab** | **Ultra-Orthodox** | **General** |
| **Date** | **Sectors** | **Sector** | **Sector** | **Sector** |
|  |  |  |  |  |
| 17-Dec-20 | 0% | 0% | 0% | 0% |
| 24-Dec-20 | 1% | 1% | 1% | 1% |
| 31-Dec-20 | 4% | 4% | 4% | 4% |
| 7-Jan-21 | 9% | 8% | 7% | 9% |
| 14-Jan-21 | 11% | 10% | 9% | 12% |
| 21-Jan-21 | 16% | 13% | 13% | 17% |
| 28-Jan-21 | 22% | 18% | 19% | 24% |
| 4-Feb-21 | 31% | 22% | 26% | 34% |
| 11-Feb-21 | 40% | 28% | 34% | 45% |
| 18-Feb-21 | 51% | 39% | 43% | 56% |
| 25-Feb-21 | 60% | 52% | 51% | 65% |
| 4-Mar-21 | 66% | 59% | 56% | 70% |
| 11-Mar-21 | 69% | 63% | 60% | 72% |
| 18-Mar-21 | 71% | 67% | 62% | 74% |
| 25-Mar-21 | 72% | 69% | 64% | 76% |
| 31-Mar-21 | 73% | 70% | 64% | 76% |

Table 4

Population by sector and age group, as of March 31, 2021
(in thousands)

|  | **Total - All** | **Arab** | **Ultra-Orthodox** | **General** |
| --- | --- | --- | --- | --- |
| **Date** | **Sectors** | **Sector** | **Sector** | **Sector** |
|  |  |  |  |  |
| Age 20+ | 5,386 | 744 | 806 | 3,836 |
|  |  |  |  |  |
| Age 20-39 | 2,273 | 376 | 382 | 1,515 |
| Age 40-59 | 1,748 | 252 | 240 | 1,256 |
| Age 60+ | 1,365 | 116 | 184 | 1,065 |
|  |  |  |  |  |
