# Supplementary material for: Addressing vaccine hesitancy and access barriers to achieve persistent progress in Israel’s COVID-19 vaccination program
Source: Isr J Health Policy Res. 2021 Aug 2;10:43. doi: 10.1186/s13584-021-00481-x (PMC8326649; doi:10.1186/s13584-021-00481-x)
Supplement: Supplementary file 2 — Additional file 2: Supplemental file 2. – tables with data on uptake of second dose. [file 13584_2021_481_MOESM2_ESM.docx]

**Supplemental file 1 – tables with data on uptake of second dose**

| **Table 1** |  |  |  |  |  |
| --- | --- | --- | --- | --- | --- |
| **Cumulative percent vaccinated with second dose, by date and age group** | | | | | |
|  | |  |  |  |  |
|  |  |  |  |  |  |
| **Date** | **Age 20+** | **Age 20-39** | **Age 40-59** | **Age 60+** |  |
|  |  |  |  |  |  |
| 24-Dec-20 | 0% | 0% | 0% | 0% |  |
| 31-Dec-20 | 0% | 0% | 0% | 0% |  |
| 7-Jan-21 | 0% | 0% | 0% | 0% |  |
| 14-Jan-21 | 3% | 1% | 2% | 8% |  |
| 21-Jan-21 | 15% | 4% | 9% | 40% |  |
| 28-Jan-21 | 28% | 8% | 22% | 67% |  |
| 4-Feb-21 | 33% | 11% | 30% | 74% |  |
| 11-Feb-21 | 40% | 15% | 42% | 77% |  |
| 18-Feb-21 | 46% | 21% | 51% | 80% |  |
| 25-Feb-21 | 52% | 30% | 57% | 82% |  |
| 4-Mar-21 | 58% | 39% | 62% | 84% |  |
| 11-Mar-21 | 64% | 49% | 67% | 86% |  |
| 18-Mar-21 | 71% | 58% | 72% | 88% |  |
| 25-Mar-21 | 74% | 62% | 75% | 90% |  |
| 31-Mar-21 | 75% | 65% | 76% | 90% |  |

| **Table 2** |  |  |  |  |
| --- | --- | --- | --- | --- |
| **Cumulative percent vaccinated with second dose, by date and sector** | | | |  |
| **Persons age 60+** | |  |  |  |
|  |  |  |  |  |
|  | **Total – All** | **Arab** | **Ultra-Orthodox** | **General** |
| **Date** | **Sectors** | **Sector** | **Sector** | **Sector** |
|  |  |  |  |  |
| 24-Dec-20 | 0% | 0% | 0% | 0% |
| 31-Dec-20 | 0% | 0% | 0% | 0% |
| 7-Jan-21 | 0% | 0% | 0% | 0% |
| 14-Jan-21 | 8% | 3% | 8% | 9% |
| 21-Jan-21 | 40% | 21% | 36% | 43% |
| 28-Jan-21 | 67% | 44% | 62% | 70% |
| 4-Feb-21 | 74% | 53% | 69% | 77% |
| 11-Feb-21 | 77% | 58% | 72% | 80% |
| 18-Feb-21 | 80% | 63% | 75% | 83% |
| 25-Feb-21 | 82% | 66% | 77% | 85% |
| 4-Mar-21 | 84% | 69% | 79% | 86% |
| 11-Mar-21 | 86% | 73% | 81% | 88% |
| 18-Mar-21 | 88% | 78% | 84% | 90% |
| 25-Mar-21 | 90% | 80% | 85% | 92% |
| 31-Mar-21 | 90% | 81% | 86% | 92% |

| **Table 3** |  |  |  |  |
| --- | --- | --- | --- | --- |
| **Cumulative percent vaccinated with second dose, by date and sector** | | | |  |
| **Persons age 20-39** | |  |  |  |
|  |  |  |  |  |
|  | **Total - All** | **Arab** | **Ultra-Orthodox** | **General** |
| **Date** | **Sectors** | **Sector** | **Sector** | **Sector** |
|  |  |  |  |  |
| 24-Dec-20 | 0% | 0% | 0% | 0% |
| 31-Dec-20 | 0% | 0% | 0% | 0% |
| 7-Jan-21 | 0% | 0% | 0% | 0% |
| 14-Jan-21 | 1% | 1% | 1% | 1% |
| 21-Jan-21 | 4% | 3% | 3% | 4% |
| 28-Jan-21 | 8% | 7% | 7% | 9% |
| 4-Feb-21 | 11% | 9% | 9% | 12% |
| 11-Feb-21 | 15% | 13% | 12% | 16% |
| 18-Feb-21 | 21% | 17% | 17% | 23% |
| 25-Feb-21 | 30% | 21% | 24% | 33% |
| 4-Mar-21 | 39% | 26% | 32% | 44% |
| 11-Mar-21 | 49% | 36% | 41% | 54% |
| 18-Mar-21 | 58% | 48% | 49% | 63% |
| 25-Mar-21 | 62% | 55% | 53% | 67% |
| 31-Mar-21 | 65% | 58% | 55% | 69% |

Table 4

Population by sector and age group, as of March 31, 2021
(in thousands)

|  | **Total - All** | **Arab** | **Ultra-Orthodox** | **General** |
| --- | --- | --- | --- | --- |
| **Date** | **Sectors** | **Sector** | **Sector** | **Sector** |
|  |  |  |  |  |
| Age 20+ | 5,386 | 744 | 806 | 3,836 |
|  |  |  |  |  |
| Age 20-39 | 2,273 | 376 | 382 | 1,515 |
| Age 40-59 | 1,748 | 252 | 240 | 1,256 |
| Age 60+ | 1,365 | 116 | 184 | 1,065 |
